# Necrotizing pneumonia requiring prolonged extracorporeal membrane oxygenation: Pushing the boundaries in pediatric ECMO

**DOI:** 10.1002/ccr3.5973

**Published:** 2022-07-11

**Authors:** Pravin R. R., Suresh Chandran, Yi Hua Tan, Biju Thomas, Jan Hau Lee, Anuradha P. Menon, Kim Kiat Ong, Arun Kumar Pugalenthi

**Affiliations:** ^1^ General Pediatrics KK Women's & Children's Hospital Singapore; ^2^ Yong Loo Lin School of Medicine National University of Singapore Singapore; ^3^ Neonatology KK Women's & Children's Hospital Singapore; ^4^ Duke‐NUS Medical School National University of Singapore Singapore; ^5^ Lee Kong Chian School of Medicine Nanyang Technological University Singapore; ^6^ Pediatric Respiratory Medicine KK Women's & Children's Hospital Singapore; ^7^ Children's Intensive Care Unit KK Women's & Children's Hospital Singapore; ^8^ Cardiothoracic Surgery Service KK Women's & Children's Hospital Singapore

**Keywords:** children, ECMO, necrotizing pneumonia, pediatric

## Abstract

Extracorporeal membrane oxygenation (ECMO) is a life‐saving rescue therapy used in acute respiratory failure refractory to invasive mechanical ventilation. Recent studies on positive outcomes of extended ECMO therapy are promising. We describe a case of a 2‐year 8‐month‐old female child with necrotizing pneumonia secondary to *Streptococcus pneumoniae*, *Influenza A*, and *Mycoplasma pneumoniae*, who survived with intact neurological function and no long‐term adverse outcomes after a prolonged ECMO run of 86 days. To the best of our knowledge, this is one of the longer durations of ECMO with transplant‐free survival in a pediatric patient requiring respiratory support with good recovery and a good functional outcome. Allowing time for native lung recovery is pivotal for optimal recovery, despite significant lung injury due to the underlying disease process. With evolving ECMO experience, clinicians may need to re‐consider the conventional maximum duration of ECMO in children with severe respiratory failure on a case‐by‐case basis.

## Graphical abstract

Use of prolonged extracorporeal membrane oxygenation (ECMO) to allow native lung recovery can result in positive outcomes for pediatric patients, although clinicians need to monitor closely for complications inherent to ECMO. Pushing the boundaries with ECMO may do pediatric patients more benefit than harm on a case‐by‐case basis.

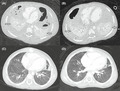

## 1 INTRODUCTION

Two million children under 5 years of age succumb annually to pneumonia globally despite vaccinations and better treatment options.^1^ Progression to respiratory failure is associated with poorer outcomes.^2^ Necrotizing pneumonia complicates up to 8% and 20% of community‐acquired pneumonia and hospital‐acquired pneumonia, respectively.^3^ Fortunately, with the advent of extracorporeal membrane oxygenation (ECMO), the survival of patients with severe respiratory failure refractory to conventional mechanical ventilation (MV) has improved over time. In specialized centers, ECMO is used in children with necrotizing pneumonia despite the precarity of associated complications such as bleeding.^3^ Prolonged ECMO use for critically ill children is a subject that has sparked much debate. While adults on prolonged ECMO have improved survival outcomes,^4^ the reverse has been documented in large‐cohort pediatric studies.^5^ Differences could be attributed to patient profiles, underlying illness, and reversibility of the disease. However, there are emerging pediatric case reports of children with necrotizing pneumonia who survive after prolonged ECMO without resection of the necrotic region.^6^ This is encouraging for clinicians who encounter such cases, as noted in our 2‐year 8‐month‐old, who had a good recovery after 86 days of ECMO support.

## 2 CASE REPORT

**FIGURE 1:**
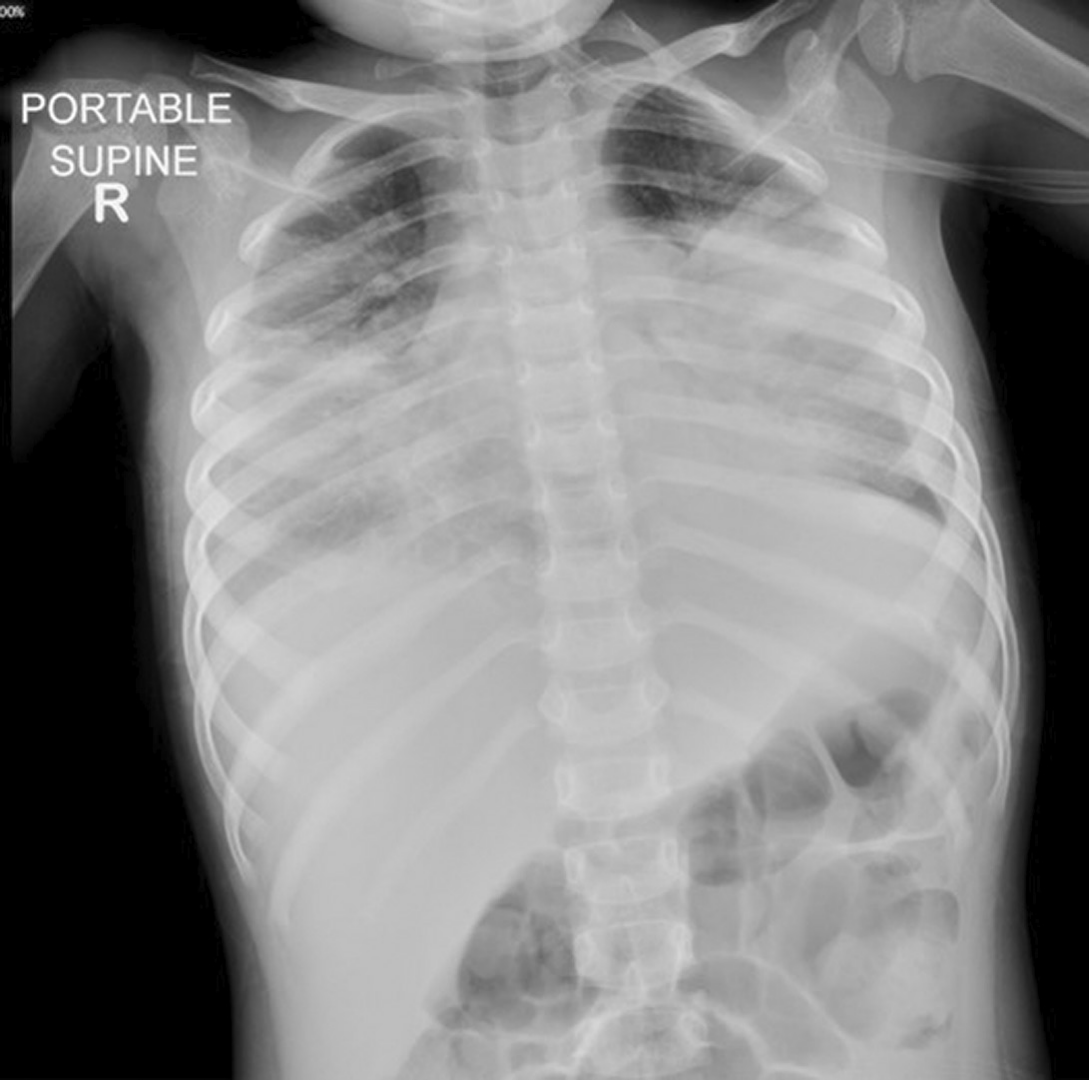
Chest radiograph at initial presentation showing bilateral consolidation and pleural effusion

A previously healthy 2‐year 8‐month‐old Indian girl presented with a 3‐day history of fever and cough. She had completed all age‐appropriate immunizations, including three doses of the 13‐valent pneumococcal conjugate vaccine. Chest radiograph showed bilateral pneumonia complicated by pleural effusion (Figure 1). Initial antimicrobial studies confirmed that she was *Influenza A* and *Mycoplasma pneumoniae* positive on the nasopharyngeal aspirate. She was treated with the following broad‐spectrum antibiotics: intravenous ceftriaxone, vancomycin, and oral clarithromycin. The pleural effusion was drained with a left‐sided chest tube. Despite pleural fluid drainage and treatment with broad‐spectrum antibiotics, she progressed to respiratory failure requiring intubation prior to transfer to our pediatric intensive care unit (PICU) on Day 8 of her illness.

**FIGURE 2:**
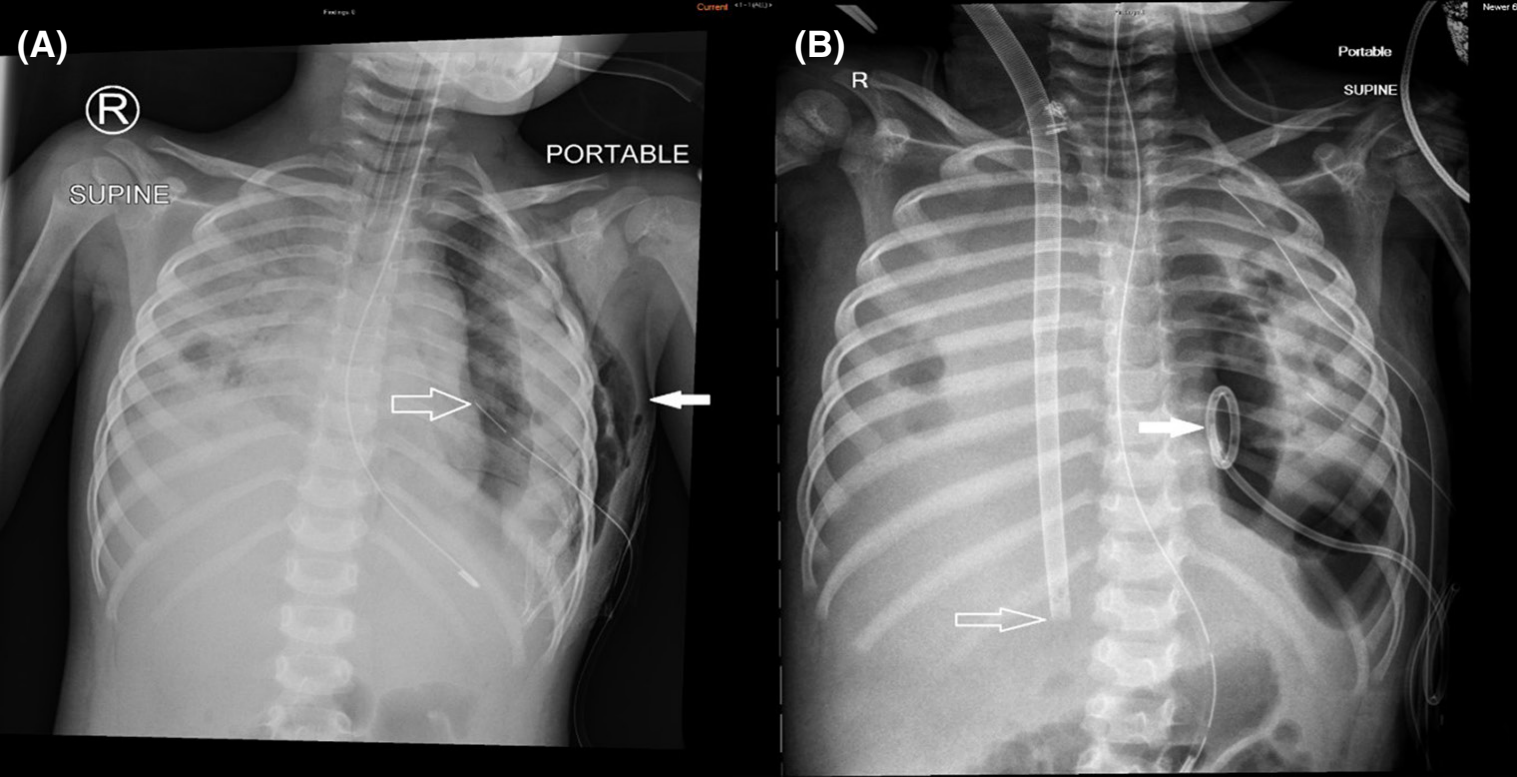
(A) Chest radiograph 4 days prior to initiation of extracorporeal membrane oxygenation (ECMO) showing bilateral consolidation, a left pneumothorax with chest drain in‐situ (open arrow) and left‐sided subcutaneous emphysema (solid arrow). (B) Chest radiograph taken on Day 6 of ECMO showing bilateral consolidation, ECMO cannula (open arrow), a coop‐loop chest drain within the left pneumothorax (solid arrow) and 2 left chest drains

Her clinical condition continued to deteriorate in our PICU. Her urine and pleural fluid tested positive for *Streptococcus pneumoniae* antigen, following which her antibiotic cover was escalated to piperacillin and tazobactam. Unfortunately, she deteriorated with repeat chest radiograph showing dense bilateral consolidation complicated by significant parapneumonic effusions (Figure 2A). Serial blood gases showed worsening type 2 respiratory failure despite high conventional MV settings, including fraction of inspired oxygen (FiO_2_) of 1.0, and persistent clinical lability leading to conversion to high‐frequency oscillatory ventilation (HFOV). Despite high settings on HFOV (mean airway pressure 23 cm H_2_O, amplitude 50, frequency 10 Hz, FiO_2_ 1.0) and a trial of nitric oxide at 30 parts per million (ppm), she remained in severe respiratory failure. Her arterial blood gas showed a pH of 7.06, partial pressure of carbon dioxide (pCO_2_) 101 mmHg, partial pressure of oxygen (pO_2_) 66 mmHg, base excess (BE) of minus 2.1 mmol/L and lactate of 0.84 mmol/L with an oxygenation index (OI) of 34.8, in keeping with severe acute respiratory distress syndrome (ARDS).^7^

Given her rapid progression of severe acute respiratory failure refractory to conventional MV, she was commenced on venovenous (VV) ECMO treatment with a size 23 French Avalon cannula via the right internal jugular vein (IJV). Post‐cannulation, ventilator settings were weaned to minimal to facilitate lung recovery. Minimal radiologic improvement in lung aeration on Day 3 of admission with poor tidal volumes indicated a suboptimal functional improvement (Figure 2B).

**FIGURE 3:**
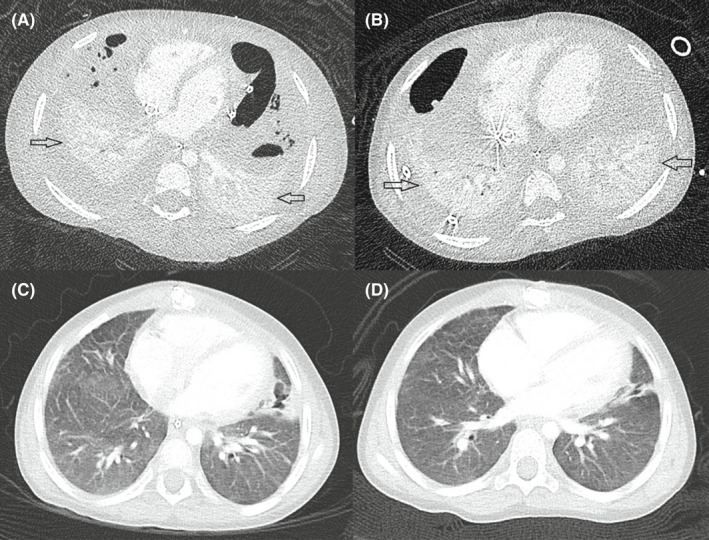
(A) CT Thorax done on Day 7 of extracorporeal membrane oxygenation (ECMO) shows bilateral collapse and consolidation (arrows). (B) CT Thorax on Day 37 of ECMO shows bilateral collapse and consolidation (arrows). (C and D) CT Thorax 2 months and 7 months post‐ECMO, respectively, showing significant improvement with minimal residual parenchymal changes

First computed tomography (CT) scan of the thorax done on Day 7 of admission showed bilateral pulmonary consolidation, likely abscess on the right, and necrotizing pneumonia on the left, with dissection into bilateral pleural spaces, suggesting bilateral empyema (Figure 3A). A left chest drain was inserted under fluoroscopic guidance to drain the empyema. A course of intravenous methylprednisolone 1 mg/kg Q12 hourly for treatment of ARDS was given for 5 days with no clinical improvement. An immunodeficiency workup, comprising of immunoglobulins, B and T lymphocyte subsets, nitro blue tetrazolium test and human immunodeficiency virus screen, was also done given the severity of the episode. All tests returned negative.

Between Days 5 and 54 of admission, she underwent six ECMO circuit changes. These were related to fibrin clot formation and bubbles in multiple sites of the circuit, mechanical issues such as movement of the cannula resulting in change of depth, and air embolism. Her circuit changes were complicated by hemodynamic instability requiring inotropic support and short periods of cardio‐pulmonary resuscitation. A repeat CT of the thorax performed on Day 33 of admission (Figure 3B) showed some improvement in previous bilateral pleural collections, but both lungs remained collapsed. There was persistent re‐accumulation of loculated pneumothoraces, suggestive of probable bronchopleural fistula, which was conservatively managed with chest tube drainage.

Given the absence of multiorgan dysfunction (serial 2D‐echocardiograms while on ECMO which showed normal cardiac function and a structurally normal heart, no evidence of pulmonary hypertension, no significant liver and renal impairment) and CT scan of the brain showing no intracranial bleed on Day 62 of her ECMO run (despite prolonged heparin use on ECMO), an extensive discussion with the family was undertaken regarding the appropriateness of continued ECMO support. This was to reinforce that there were no further options of pediatric lung transplant offered in our center and the high risk of complications with continuation of ECMO. A consensus on continuing ECMO was reached between the medical team and parents given the potential for her lung recovery with time. Furthermore, a decision was made to terminate the ECMO if she developed any irreversible organ injury or intracranial complications.

A trial off VV ECMO decannulation was attempted on Day 69 of her ECMO run, which she failed after she developed significant desaturations. On Day 76 of her ECMO run, she was converted to venoarterial (VA) ECMO via a right femoral arterial cannulation. Venous cannulation was done through the right atrium as the right femoral vein was of inadequate caliber to allow for full advancement of the venous cannula to provide adequate flow. The decision to convert to VA ECO was made firstly due to inability to wean off ECMO in view of high ventilatory settings, and secondly in view of highly friable neck tissue over the internal jugular veins on the right neck due to prolonged use as well as lack of other suitable sites for venous cannulation owing to size and central line placement. Moreover, during the last circuit change on Day 71 of ECMO, she desaturated to 0% and required 5 min of cardio‐pulmonary resuscitation, indicating that her reserves were poor and making her hemodynamic status tenuous. While we acknowledge that conversion from VV to VA ECMO at this juncture is unusual, our case highlights the challenge with pediatric ECMO cannulation in prolonged runs.

In the days following VA ECMO cannulation, she was noted to have significant radiological improvement in lung aeration and tidal volumes (up to 4 ml/kg). She was successfully decannulated on Day 10 of VA ECMO to conventional MV, completing a total of 86 days of ECMO. Before decannulation, she underwent two flexible bronchoscopies, bronchoalveolar lavage for pulmonary toileting and intensive chest physiotherapy to optimize lung recruitment.

Post‐decannulation of ECMO, she remained on conventional MV. Apart from an inferior vena cava (IVC) thrombus requiring subcutaneous enoxaparin treatment, she did not have any other ECMO‐related complications. She remained sedated for the entire duration of the ECMO run, requiring prolonged infusions of benzodiazepines and opioids owing to movement of the neck cannula and frequent complications of air entrainment.

**FIGURE 4:**
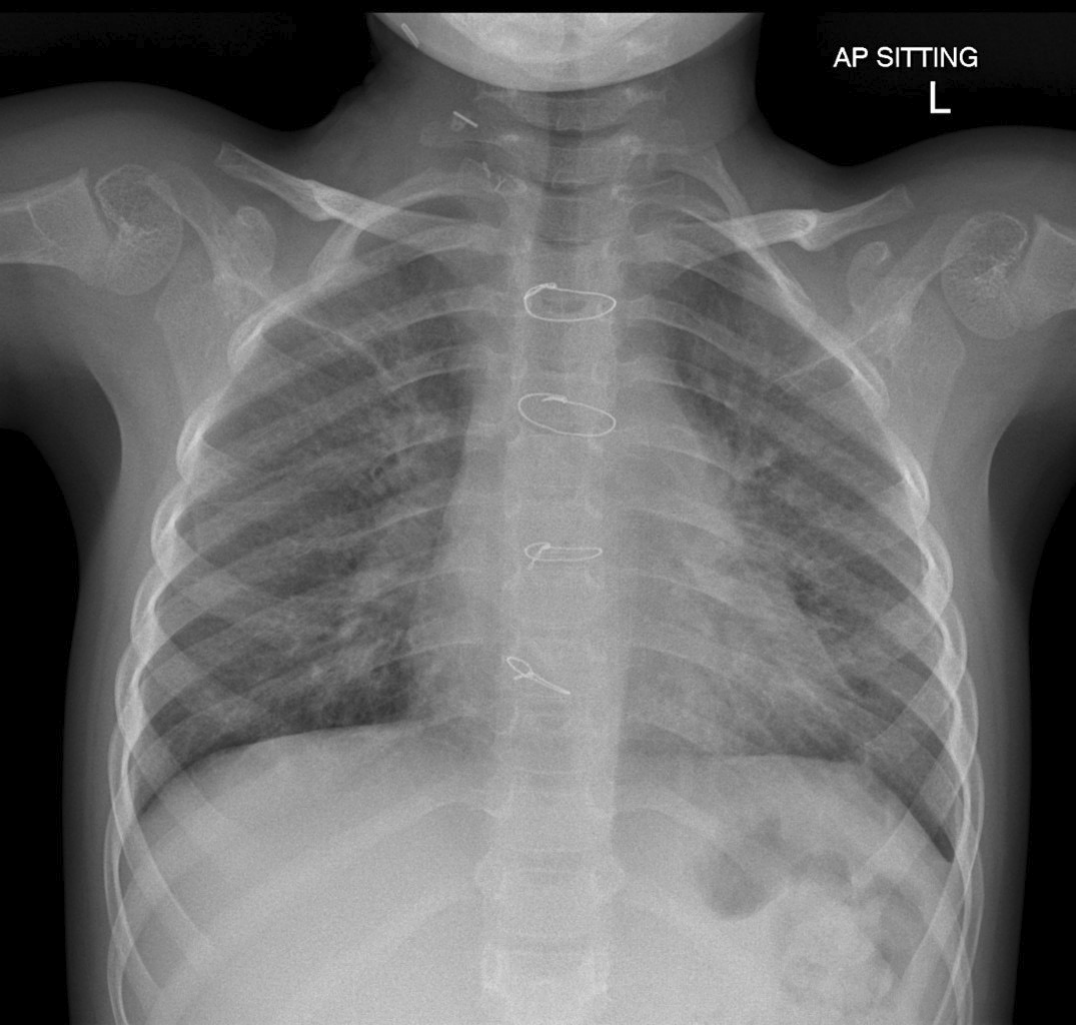
Chest radiograph performed post‐decannulation of tracheostomy 3 months post‐discharge

Given the likely need for long‐term invasive ventilatory support, she underwent a tracheostomy. After tracheostomy on Day 96 of PICU admission, she was commenced on bi‐level positive airway pressure (BiPAP) ventilation for 12 days and subsequently converted to continuous positive airway pressure (CPAP) of +7 cm H_2_O on room air as her respiratory status improved. She was allowed periods of de‐venting with the physiotherapist. CT thorax performed 2 months post‐ECMO (Figure 3C) showed remarkable improvement compared with the CT thorax done on Day 41 of her ECMO run (Figure 3B). She remained on CPAP via tracheostomy till 3 months after discharge when she was completely weaned from CPAP. A follow‐up CT thorax (Figure 3D) done 4 months after discharge showed mild airspace opacification in the apical segment of the right upper lobe, linear scarring, and atelectasis in the middle lobe and lingula. Subsequently, she underwent an uneventful decannulation of tracheostomy at 3 years 6 months of age. An overnight oximetry performed post‐decannulation of tracheostomy, showed a mean SpO_2_ of 98%, and her morning capillary blood gas showed a pH of 7.45 and pCO2 35.4 mmHg. A chest radiograph done 3 months post‐decannulation showed a residual linear band of atelectasis in the right upper lobe and non‐specific mild changes throughout bilateral lung parenchyma (Figure 4).

After recuperating from her prolonged illness, she resumed school and regular physical activities a year after discharge. At her most recent outpatient review in the respiratory clinic 37 months 20 days after her PICU admission, there were no reported respiratory symptoms with exertion. No spirometry test was performed yet given the age.

## 3 DISCUSSION

Since 1993, ECMO survival trends for pediatric patients with acute respiratory failure, without any underlying comorbidities, have been improving.^8^ The usual ECMO duration is 7–10 days, while a duration >14 days is considered as prolonged ECMO.^6^ It is well established that a shorter ECMO duration is associated with lower morbidities and mortality.^9^ However, prolonged duration of ECMO has been increasingly reported across various studies^5^, ^10^, ^11^, ^12^, ^13^ including ours. As the decision for prolonged ECMO is challenging, the physician should weigh the risks and benefits of awaiting native lung function to recover with a possibility that it may not, versus the risks of complications such as intracranial hemorrhage, bleeding, and infection. Studies on children requiring prolonged ECMO for respiratory failure show a step‐wise decrease in survival rates over time: 30% and 27% after 4 weeks and 45 days of ECMO, respectively.^10^

Our patient survived after 86 days of combined VV and VA ECMO, which is one of the most prolonged pediatric ECMO runs with a favorable outcome in terms of a transplant‐free survival, in a center which does not offer transplant. The longest documented duration is 394 days in a 13‐year‐old awaiting cardiac transplantation in 2021, who eventually received a cardiac transplant.^13^

Positive prognostic factors specific to this case were the absence of pre‐existing comorbidities, female gender, absence of multi‐organ failure,^14^ and no intracranial bleeds noted on CT brain scans done while the patient was on ECMO. Moreover, she was initiated on ECMO within 3 days of MV. Studies have shown that ECMO initiation after 2 weeks of MV is a poor prognostic factor due to pre‐existing ventilator‐induced lung injury. Other clinical factors associated with poor outcomes include male gender and pre‐ECMO respiratory acidosis.^8^

The nature of underlying disease process also affects outcomes of prolonged ECMO as the rate of lung recovery will be faster in patients with ARDS secondary to viral pneumonia or pulmonary hypertension compared with ARDS secondary to complications of malignancies or associated treatment.^4^ Besides patient factors, outcomes of prolonged ECMO are also influenced by skills and experience of the ECMO team and advances in ECMO circuit technologies, which have been improving over time. Cannulation strategies in terms of choice of VV and VA ECMO are important and depend on the suitability of the patient and risk for complications on therapy. She had a total of 8 circuit changes and frequent circuit changes are associated with higher mortality.^15^

Our case also enables clinicians to reflect on the need for surgery and lung transplant for patients with significant lung destruction secondary to necrotizing pneumonia. These procedures carry their innate complications. Giving a longer duration for the patient's lung to recover is important, although it is hard to predict the exact duration needed, as it is often patient‐dependent. Lung recovery strategies such as regular chest physiotherapy and secretions management also play an often‐underestimated pivotal role in helping the lungs recover.^12^

For complex cases such as our patient requiring prolonged ECMO support, there can be a myriad of factors affecting ECMO outcome including patient factors such as age and gender, underlying primary diagnosis and disease related as well as treatment related factors, duration of MV, advancements in ECMO technologies and institution‐based expertise.^8^ In patients with long runs of ECMO, there are multiple challenges encountered along the way as illustrated in our case report ranging from mechanical complications, cannulation complications and challenges with sedation and venous access. Significant challenges were encountered with sedation given prolonged ECMO run in a patient of such a young age with inherent risks of developing tolerance and side effects from the agents used. A cohesive ECMO team that is well‐trained to deal with the quotidian challenges of a patient on long‐term ECMO cannot be understated. Managing the constant circuit changes above the other complications of a long ICU stay, is not an easy undertaking. In our case, she only had single organ dysfunction but in other cases, there may be multiple organ dysfunction, carrying a guarded prognosis.

On a case‐by‐case basis, the team has to be prepared to deal with the issues with regular and open conversations with family and relevant stakeholders involved such as the cardiothoracic surgeons, cardiology, and respiratory teams. Continued engagement with the family to ensure a shared decision‐making process in challenging patients, for whom there is a lack of robust evidence‐based protocols to guide key decisions such as duration of or discontinuation of ECMO, at regular critical intervals are important in contributing to better outcomes for these children.^5^

Thankfully, this child had a good functional outcome owing to the cohesive ECMO team comprising of doctors, nurses and allied health, the collaboration between multiple subspecialties and the unwavering faith of the family. Neurological examinations done at her follow‐up visit were normal although no formal neurodevelopmental test has been performed yet.

## 4 CONCLUSION

Further large‐cohort studies analyzing mortality outcomes or validated scoring systems of children receiving prolonged ECMO for acute respiratory failure, particularly necrotizing pneumonia, would help guide clinical decision‐making for intensivists in terms of duration of therapy and endpoints of treatment. Our experience suggests that prolonged use of ECMO may be justified in children with pediatric ARDS resulting from severe necrotizing pneumonia in the absence of significant comorbidities and multiorgan dysfunction. However, one has to acknowledge that there are challenges associated with a long ECMO run and this has to be applied on a case‐by‐case basis.

## AUTHOR CONTRIBUTIONS

PRR, SC, YHT, BT, JHL, APM, OKK, and AKP contributed to the conception of this report, revised the manuscript critically, approved its final version. PRR was responsible for drafting the manuscript.

## CONFLICT OF INTEREST

All authors declare that no conflict of interest exists.

## CONSENT

Written informed consent was obtained from the patient's parents to publish this report in accordance with the journal's patient consent policy.

## Data Availability

Data available on request from the authors.
